# Challenges Faced in Engaging American Indian Mothers in an Early Childhood Caries Preventive Trial

**DOI:** 10.1155/2015/179189

**Published:** 2015-05-19

**Authors:** Tamanna Tiwari, Judith Albino, Terrence S. Batliner

**Affiliations:** Centers for American Indian and Alaska Native Health, Colorado School of Public Health, University of Colorado Anschutz Medical Campus, Aurora, CO 80045, USA

## Abstract

*Objective*. This study explores the challenges faced by the research implementation team in engaging new mothers in a community oral health prevention intervention in an American Indian (AI) reservation community. *Methods*. Qualitative methods in the form of in-depth interviews were used in the study. Qualitative data were collected from research staff workers at a field site, who were involved in the implementation of a culturally tailored, randomized controlled trial of a behavioral intervention utilizing Motivational Interviewing (MI). *Results*. Several challenges were described by the field staff in engaging new mothers, including low priority placed on oral health, lack of knowledge, and distractions that reduced their ability to engage in learning about oral health of their child. Other difficulties faced in engaging the mothers and the AI community at large were distrust related to racial differences and physical and environmental barriers including poor road conditions, lack of transportation and communication, and remoteness of data collection sites. The field staff developed and applied many strategies, including conducting home visits, applying new communication strategies, and interacting with the community at various venues. *Conclusion*. Prevention interventions for ECC need to target AI mothers. Strategies developed by the field staff were successful for engaging mothers in the study.

## 1. Introduction

Mothers play an important role in determining the oral health of their children. Maternal oral health knowledge, behaviors and beliefs such as feeding practices, oral hygiene maintenance for the child, failure to access professional dental care, and the beliefs that a child is not susceptible to caries or that primary teeth are not important are associated with early childhood caries (ECC) [1]. These maternal beliefs and oral health practices appear to place a child at greater risk of developing dental caries [1]. Also, mothers' oral health status is a strong predictor of the oral health status of their children [2].

ECC is a multifactorial disease involving biological, microbial, and behavioral factors [3] and the three etiological factors must simultaneously be present for initiation and progression of the disease. There is strong evidence that* S*.* mutans* and lactobacilli are the primary agents involved in the development of caries in both children and adults [4]. Studies using genetic/molecular techniques have shown that the source of the* S. mutans* infection in children is, predominantly although not exclusively, attributed to vertical transmission from the mothers [5]. High levels of* S. mutans* andlactobacilli in the mother's mouth contribute to maternal transfer as do maternal dietary habits and poor oral hygiene [6]. Maternal behaviors such as tasting the infant's food and sharing utensils can transmit bacteria from the mother to the child [6].

Maternal psychosocial factors, including stress, oral health beliefs, fatalistic attitudes, and cultural factors, are associated with ECC and with dental health services utilization [1, 7–9]. Other characteristics such as maternal oral health literacy, self-efficacy, and ethnic and cultural-specific beliefs about oral health can be contributing factors to ECC [9, 10]. Because a child's oral health is largely dependent on maternal biological and psychosocial factors, ECC becomes a women's health issue, making it imperative to engage mothers in behavior change methods beginning at a child's birth, or even prenatally.

American Indian (AI) children have the highest rate of ECC of any population group within the United States [11]. American Indian mothers living on reservations often are disproportionately burdened with the social disadvantages associated with unemployment, low levels of education, and poverty [12]. These environmental factors may contribute to maternal psychological distress and, along with other factors such as low self-efficacy, social support, and inadequate preventive health services, can place AI children at still higher risk of ECC. Our data have demonstrated that AI parents who do not adhere to recommended oral health behaviors also report more chronic distress than others [13]. They also had poor oral health knowledge and demonstrated external locus of control, suggesting little confidence in their ability to influence their children's oral health [13]. In fact, the oral health of these AI children was significantly worse than that of children whose parents had greater oral health knowledge and more positive attitudes [14]. These data suggested that working to improve maternal behavior, knowledge, and attitudes might lead to improved oral health in AI children.

An oral health prevention trial for behavior change using Motivational Interviewing (MI) in AI mothers is underway in a Northern Plains tribal region. We speculated that when mothers learn more about oral health during MI sessions and engaged in supportive discussions about the importance of oral health practices and appropriate action steps, they will be better able to formulate oral health care goals for their children and develop the confidence and motivation needed for behavior change. However, several challenges were faced by investigators and research field staff in recruiting and engaging these mothers in the study. This paper describes the challenges and barriers faced by the field staff for an oral health prevention trial as they worked to engage AI mothers living in rural and remote locations. We also discuss the strategies developed as these staff worked to overcome the challenges they faced.

## 2. Materials and Methods

In 2008, the Center for Native Oral Health Research at the University of Colorado Anschutz Medical Center was funded by the National Institute for Dental and Craniofacial Research (U54DE019259) to conduct a randomized controlled trial to determine whether a behavioral intervention using MI with AI new mothers will achieve a greater reduction of caries experience in children younger than three years than would enhanced community services alone [15]. Six hundred mothers and their newborns were enrolled and randomized to one of two groups over two years, with a period of follow-up lasting until the child's third birthday. The trial has been approved by the Tribal Research Review Board and the Colorado Multiple Institutional Review Board.

This study was a qualitative evaluation of the challenges faced by the field staff in engaging AI mothers in the prevention trial; as such, it falls under the purview of quality assurance and management of data collection for the trial and is not subject to IRB review, other than what is required for the clinical trial. The authors created a semistructured interview guide and conducted in-depth interviews, lasting 40–50 minutes each, with the five field staff members, who carried out the intervention. All interviews were digitally recorded and later transcribed. Verbal consent was obtained from the staff members who participated in the interviews, and participation was voluntary. Staff members were requested not to identify other staff members or themselves during the interviews to maintain confidentiality. All digital recordings are stored on a secure server. Following standard qualitative analysis procedures [16, 17], all the transcripts were coded. After initial coding was completed, codes were iteratively refined as various themes and subthemes emerged as shown as follows.

*Challenges.* They include the following.

 Engaging the mothers:
   low priority,
   lack of knowledge,
   distractions.
 Balancing potential conflicts between research policy and cultural norms.
 Not being American Indian.
 No gender based challenges.

*Barriers.* They include the following.

 Physical and environmental barriers:
   poor road conditions,
   lack of transportation and communication,
   remoteness of data collection sites.

*Strategies*. They include the following.

 Research implementation which is a teamwork.
 Conducting home visits.
 New communication methods.
 Interacting with the community at various venues.
 Culturally sensitive strategies.

## 3. Result and Discussion

### 3.1. Results

Three major themes and several subthemes emerged from the transcripts as shown above. The field staff described several challenges they faced in implementing the research, particularly those related to engaging new mothers in the study and the MI intervention. The staff pointed out that some mothers living on the reservation placed low priority on the oral health of their children, thus making it difficult to recruit them into the study. Reflecting on the fact that AI families living on the reservation often have to deal with unemployment, food insecurity, and problems related to inadequate housing and transportation, they expressed empathy and understanding that oral health often was not the highest priority for some of these mothers.

### 3.2. Comments of the Staff

Staff comment:

*Mothers deal with so many things here, they want to participate in something like this, they want the best for their children but it is almost impossible.*

The field staff described that AI mothers had relatively little knowledge about oral health; for instance, they often did not have knowledge about the importance of primary teeth or about the causes of dental caries. Moreover, the main source of their knowledge was their mothers and extended family.

Staff comment:

*I think my biggest challenge is ignorance; they just do not know. And the other challenge is to get them to think differently than their mothers and grandmothers.*

Pointing out that many study visits are done in the participants' homes, staff noted that the mothers often were distracted by older children or by household chores and could not always fully engage with the staff who were there for an MI session or for conducting the dental assessment for the child.

Staff comment:

*The main challenge that I face in engaging new mothers is distractions, mainly their other older kids who want attention.*

Though all the staff are from the community, some of them are not American Indian, and this sometimes makes it difficult to create trust and thereby makes the job of recruiting mothers more difficult.

Staff comment:

*I think it is also different for me because I am a Caucasian person, I am not from here and for me it is different to come in here, I am the outcast here, I am the different person here.*

All the field staff members are women, and they indicated that they have not faced any gender based challenges or barriers in engaging the mothers in the study. In fact, they said that being a woman was helpful in building rapport with the study participants. They believed that participants felt safer and more comfortable inviting women into their homes and sharing their stories with them.

Staff comment:

*I always get positive responses from mothers as I am a mom too. I think mothers are more attentive to other women.*

The staff described some physical and environmental barriers they encountered that prevented them from being more productive in improving participant engagement in the study. They spoke about the vastness and remoteness of the reservation, poor road conditions, inclement weather, and poor communication methods—all of which could be overwhelming and delay the process of reaching out to study participants. They thought that not reaching the participants at a given time could send the wrong signals related to their role in helping new mothers in reaching oral health goals for their child.

Staff comments:

*I guess the number one difficulty is that everything is so spread out and so far away. You have to have a vehicle to survive around here.*

*Just access, whether we are going to them or they (study participants) are coming to us. Anytime it rains or snows it reduces our ability to reach to them as the roads are muddy.*

Despite all the challenges they have faced, the staff have developed innovative ways to engage the mothers and the AI community at large in this oral health prevention trial. They informed us in the interviews that they are using new communication strategies such as Facebook and sending postcards to interact and engage new mother on the reservation.

Staff comment:

*People have no phone, no car, but they have Facebook 24/7.*

They said they interact with the mothers at various community events, health clinics, and after-school programs for the children. This has not only provided ways to gain the trust of the community and the study participants but also increased their interest in oral health in general. They said they try to be culturally sensitive and are adapting to the local culture.

Staff comment:

*When I call them I talk to them as if they are my friends on an everyday basis rather than clients or a participant in the study.*

### 3.3. Discussion

Results from this qualitative assessment reveal a number of difficulties of engaging AI mothers in an oral health intervention for their children, even though AI children have the highest need for such preventive interventions. One of the challenges is to prioritize the child's oral health for AI mothers and to increase their acceptance of such interventions. Though there are many physical and environmental barriers in reaching out to these mothers, the most salient challenge is to get them interested in the prevention of oral disease and then to help them develop an understanding of oral health issues and the steps they can take to prevent oral disease in their children. The use of Motivational Interviewing (MI) in this trial is helping to prioritize the oral health of children for the participating mothers and the families. MI is complementary to the cultural values of AI/AN people; it emphasizes listening, learning, and respect, and it creates space to include spirituality and religious practices, all of which are essential to engage and elicit behavior change in AI mothers [15]. Previous studies have recommended including oral health in anticipatory guidance during prenatal visits for indigenous women and using community-based promotion initiatives to emphasize the importance of oral health for pregnant women and their infants, which might help to prioritize oral health in all indigenous children since birth [18].

Data from this study and the previous work reported in the research literature have shown that maternal physical and psychosocial health and oral health knowledge are associated with oral health in their children, or with oral health behavior on behalf of their children. For example, mother's self-efficacy is strongly associated with frequency of brushing child's teeth [5]. A number of false beliefs have been found, including the idea that primary teeth are not important because they fall out and the child will get a “second chance” with the permanent teeth [19]. We also have found that, in AI populations, oral health decisions for children often are made by multiple members of the family, and some of those family members may discourage preventive oral health practices and support accessing dental care only when there is a serious problem.

Research staff in the study are overcoming the barriers of conducting research in remote and rural settings and developing successful strategies to engage new mother in oral health interventions by gaining trust of the community and being sensitive to local norms. These include conducting home visits, applying new communication strategies (Facebook), and interacting with the community at various venues. By providing flexibility to accommodate the work and childcare schedules of the study participants, conducting home visits, and participating in community events and pow wows, the field staff not only have gained the trust of the participants and the community but also have been able to spread awareness about oral health in general. Involving the AI communities as active members of research and adapting engagement approaches according to community or tribal feedback are some of the successful strategies reported in the literature [20, 21].

These approaches were welcomed by the mothers and the AI community more generally and suggest that interacting with mothers, who are the main gatekeepers for a child's oral health, targeting behavior change in them, and building capacity at the family and community level could reduce ECC in AI children. Future oral health prevention interventions can utilize these tested strategies and approaches to successfully engage mothers and increase oral health awareness for the family at large.

## 4. Conclusion

Clearly, the prevention of ECC needs to target AI mothers; improving their oral health knowledge and decision making capacity could bring about family-level and community-level changes in preventing ECC.
